# Phase-Space Approach for Topological Phase Transitions in Silicene

**DOI:** 10.3390/e27080857

**Published:** 2025-08-12

**Authors:** Maciej Kalka, Piotr Pigoń, Bartłomiej J. Spisak

**Affiliations:** Faculty of Physics and Applied Computer Science, AGH University of Krakow, al. A. Mickiewicza 30, 30-059 Kraków, Poland

**Keywords:** silicene, topological phase transition, Wigner distribution function, Wigner–Rényi entropy, topological quantum number, Landau states

## Abstract

Silicene is a two-dimensional silicon monolayer with a band gap caused by relatively strong spin–orbit coupling. This band gap can be steered using a vertical electric field. In turn, the change in this electric field value leads to a transition from a topological insulator to a bulk insulator regime. This study aims to develop a phase-space approach to detecting the topological phase transitions in silicene induced by the presence of parallel magnetic and electric fields with the aid of the concept of topological quantum number based on the Wigner–Rényi entropy. A reinterpreted definition of the Wigner distribution function is employed to determine this indicator. The topological phase transition in silicene as a function of the electric field in the presence of the magnetic field is confirmed through the use of the topological quantum number determined for the one-half, Shannon and collision entropies.

## 1. Introduction

Over the years, the interest in two-dimensional materials is rapidly growing. It began with the exfoliation of graphene—a single honeycomb layer made of carbon atoms [1] which exhibits unique properties. For example, graphene combines exceptional mechanical strength with atomic-scale thickness, and its charge carriers demonstrate high mobility with zero effective mass. The electronic transport near the K and $K^{\prime }$ points (the corners of the Brillouin zone) is described with the Dirac-like equation, which indicates a linear dispersion relation [2]. Since the experimental discovery of graphene, there have been two possible ways to go. The first one was to understand and examine graphene and its modifications thoroughly, e.g., bilayer graphene, which shows the phase transition under the influence of external voltage [3], and the second focused on finding other graphene-like systems. Silicene represents one such promising system [4]—a two-dimensional honeycomb lattice composed of silicon atoms rather than carbon. Similarly to graphene, silicene is described by a linear dispersion relation near the K and $K^{\prime }$ points. However, silicene has strong spin–orbit coupling, which provides mass to Dirac fermions [5]. Due to this, they are described as massive Dirac fermions. Also, it enables the observation of various exotic phenomena, including the quantum spin Hall effect [6,7].

Moreover, silicene can exist in a topological insulator state [8] characterized by insulating behavior in the bulk while supporting conduction at the edges of the system, where topologically protected surface states appear [9]. Hence, electronic transport is viewed as dissipationless, making silicene particularly attractive for potential practical applications [10]. The topological character of a system can be determined by calculating its Chern number, an integer topological invariant, which can take a value of zero for a trivial, band insulator or non-zero integer for a topological insulator. This distinction can be used to analyze the topological phase transition in the considered systems under external perturbations due to magnetic or electric fields. In general, topological phase transition in various systems [11,12,13,14,15,16,17] are widely studied due to their potential application in electronic devices. Alternatively, topological phase transitions can be characterized using information-theoretic methods formulated in phase-space language. The aim of this study is to develop a phase-space approach to detecting the topological phase transition in silicene induced by the presence of magnetic and electric fields with the help of the concept of Wigner–Rényi entropy [18]. This innovative method analyzes pure quantum states through their Wigner distribution functions, which serve as phase-space representations of the wave functions [19,20]. Such reinterpretation of the Wigner distribution function forms an intermediate step in identifying topological states of matter through information-theoretic entropic measures rather than traditional band structure calculations.

The rest of this work is organized as follows: In Section 2, we present a mathematical model of the silicene layer perturbed by parallel magnetic and electric fields and analyze their influence on the spectral properties of the Hamiltonian. Section 3 contains a primer of the phase-space approach based on the Wigner distribution function and, resulting from this, the concept of Wigner–Rényi entropy. Further, Section 4 contains our main results, namely, the calculations of the topological quantum number based on the combined Wigner–Rényi entropy definition. The determination of this quantity was possible owing to the derivation of the exact form of the Wigner distribution function for the electron state in silicene in the presence of parallel magnetic and electric fields. Finally, the paper concludes with a summary.

## 2. Selected Spectral Properties of the Silicene Hamiltonian

Silicene is a two-dimensional silicon layer arranged in a honeycomb lattice structure which consists of two inequivalent sublattices of Si atoms denoted by the symbols *A* and *B*. Its structure is slightly buckled, with one of the two sublattices of the honeycomb lattice being displaced vertically with respect to the other [21]. This structural property of silicene and the fact of having a strong spin–orbit interaction allow one to steer the width of the band gap by applying a vertical electric field [22]. The electronic properties of this system can be derived from the effective low-energy Hamiltonian around a single corner point K (or an inequivalent point $K^{\prime }$) in the form [23]

(1) $$\hat{H}=v_{F}\left(\xi p_{x}{\hat{\tau }}_{x}+p_{y}{\hat{\tau }}_{y}\right)-\frac{1}{2}\xi s\Delta_{SO}{\hat{\sigma }}_{z}{\hat{\tau }}_{z}+\frac{1}{2}\Delta_{z}{\hat{\tau }}_{z}$$

where ${\hat{\sigma }}_{i}$ and ${\hat{\tau }}_{i}$ with i=0,x,y,z represent the identity and Pauli matrices associated with the spin and pseudospin degree of freedom of the systems, respectively. Further, $v_{F}$ is the Fermi velocity, $p=(p_{x},p_{y})$ stands for the momentum in the plane x−y, and the symbol ξ is the valley index for the two inequivalent corner points K and $K^{\prime }$ (ξ=1 for K, and ξ=−1 for $K^{\prime }$), while the symbol *s* represents spin-up (s=1) or spin-down (s=−1). The last two quantities, $\Delta_{SO}$ and $\Delta_{z}$, describe the strength of the spin–orbit coupling and the strength of the electric field perpendicular (along the *z*-axis) to the material, respectively. The standard set of parameters for silicene were used, namely, the Fermi velocity $v_{F}\approx 5\times 10^{5}$ m/s and the spin–orbit coupling $\Delta_{SO}=4$ meV [24]. Also, the strength of the electric field can be expressed as follows: $\Delta_{z}=E_{z}d$, where d≈0.46 Å is the nearest-neighbor distance.

The solution of the eigenproblem for the Hamiltonian in the form in (1) leads to the following formula for the energy spectrum:

(2) $$\epsilon_{\xi s}=\pm \sqrt{\hbar^{2}v_{F}^{2}k^{2}+\Delta_{\xi s}^{2}}$$

where $k=\sqrt{k_{x}^{2}+k_{y}^{2}}$ is the magnitude of the wave vector and the symbol $\Delta_{\xi s}$ is defined as follows:

(3) $$\Delta_{\xi s}=-\frac{1}{2}s\xi \Delta_{SO}+\frac{1}{2}\Delta_{z}.$$

**Figure 1:**
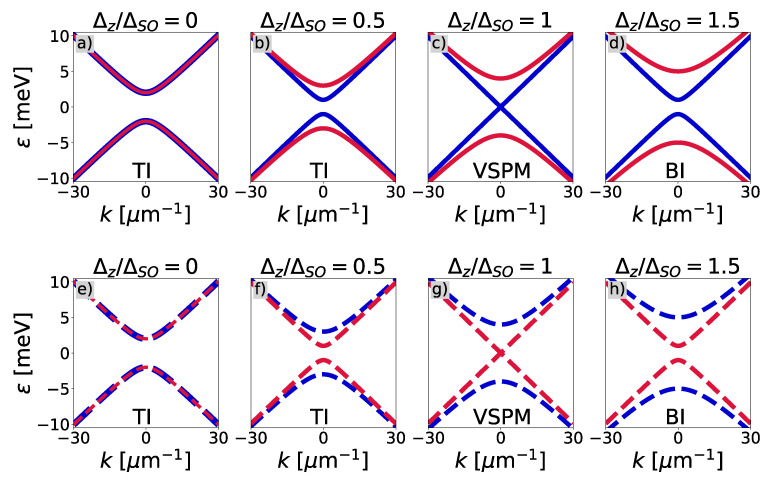
Schematic representation of the band structure evolution at the **K** (**a**–**d**) and $K^{\prime }$ (**e**–**h**) points for different ratios of the vertical electric field to the spin–orbit coupling. Red and blue lines indicate spin-up and spin-down states, respectively.

Figure 1 presents the effect of the electric field on the band structure for the spin-up and spin-down electrons (the red and the blue lines, respectively) near the K (the solid lines) and $K^{\prime }$ (the dashed lines) valleys. We considered four situations dependent on the ratio of the electric field to the spin–orbit coupling strength. Figure 1a depicts a situation without the electric field, meaning $\Delta_{z}/\Delta_{SO}=0$. The spectrum consists of doubly degenerate bands separated by the band gap, resulting from the non-zero spin–orbit coupling. Also, the spectrum is degenerated due to the valleys. The system exhibits a topological insulator state [23]. In Figure 1b, both the spin and valley degeneracy vanish due to the interplay of both the electric field and spin–orbit coupling. Two bands associated with the spin-down electrons are closer to each other, reducing the band gap width. However, the system remains in the topological insulator state. In Figure 1c, the electric field reaches the critical value, being equal to the spin–orbit coupling strength, meaning $\Delta_{z}=\Delta_{SO}$. In this case, the band gap closes, forming a Dirac cone. The system is considered a valley–spin-polarized metal (VSPM) [25]. In Figure 1d, the value of the electric field is higher than the spin–orbit coupling ($\Delta_{z}>\Delta_{SO}$). The band gap reopens. It comes to the band inversion, and the system becomes a band insulator [25]. Let us note that a band structure for the $K^{\prime }$ valley is analogous (cf. Figure 1e–h), with an interchange of bands describing electrons with spin-up and -down. Thus, changing the electric field lifts the spin and also valley degeneracy. Moreover, it leads to the closing and reopening of the band gap. The band gap closes with the critical point when the applied electric field is equal to the spin–orbit strength. Also, this critical point determines the topological phase transition between the topological and band insulator states.

According to the previous discussion, the effect of the applied vertical electric field on the silicene layer is twofold: it removes the spin and valley degeneration. These observations open up unique possibilities for investigating the role of the Landau levels in topological phase transition after applying a vertical external magnetic field to the silicene layer that is under the influence of the parallel electric field. An inclusion of the effect of the magnetic field into the electronic spectrum of the charge carriers in the silicene layer can be realized by the principle of minimum coupling with the electromagnetic field. In this case, the Peierls substitution is used in the original Hamiltonian, taking into account the Landau gauge, i.e., A=(−By,0,0). As a result, the following Hamiltonian is obtained [23]:

(4) $$\hat{H}=v_{F}\left(\xi \left(p_{x}-eBy\right){\hat{\tau }}_{x}+p_{y}{\hat{\tau }}_{y}\right)+\Delta_{\xi s}{\hat{\sigma }}_{z}{\hat{\tau }}_{z}+\Delta_{Zeeman}\left(B\right){\hat{\sigma }}_{z},$$

where the last term on the right-hand side, $\Delta_{Zeeman}\left(B\right)=g\mu_{B}B/2$ corresponds to the Zeeman term. In fact, this term can be neglected in the further analysis because its contribution to the energy spectrum of silicene is negligibly small, i.e., $\Delta_{Zeeman}/\Delta_{SO}\approx 10^{-4}$ or $10^{-5}$ for the used values magnetic fields, B=1 and 10 mT. The energy spectrum associated with the Hamiltonian in (4) in which the Zeeman term is neglected has the form

(5) $$\epsilon_{n}=\left\{\begin{array}{ccc} \mathrm{sign}\left(n\right)\sqrt{\Delta_{\xi s}^{2}+\left|n\right|\hbar^{2}\omega^{2}} & \mathrm{for} & n\neq 0\\ -\xi \Delta_{\xi s} & \mathrm{for} & n=0, \end{array}\right.$$

where $\omega =v_{F}\sqrt{2eB/\hbar }$ is the cyclotron frequency. n∈Z and labels the Landau levels; according to the adopted notation, positive numbers are reserved for electrons, whereas the negative numbers correspond to holes. In turn, for sublattices *A* and *B*, the two-component eigenfunctions $\psi_{n}\left(x,y\right)$ associated with the eigenenergies $\epsilon_{n}$ can be written in the following form:

(6) $$\psi_{n}\left(x,y\right)=\left[\begin{array}{c} -iA_{n}^{\xi s}\varphi_{\left|n\right|-\frac{1+\xi }{2}}\left(y\right)\\ B_{n}^{\xi s}\varphi_{\left|n\right|-\frac{1-\xi }{2}}\left(y\right) \end{array}\right]e^{ik_{x}x},$$

where the coefficients $A_{n}^{\xi s}$ and $B_{n}^{\xi s}$ are valley- and spin-dependent probability amplitudes have the form [23,26]

(7) $$A_{n}^{\xi s}=\left\{\begin{array}{ccc} \mathrm{sign}\left(n\right)\sqrt{\frac{|\epsilon_{n}|+\mathrm{sign}\left(n\right)\Delta_{\xi s}}{2|\epsilon_{n}|}} & \;\mathrm{for}\; & n\neq 0\\ \frac{1-\xi }{2} & \;\mathrm{for}\; & n=0, \end{array}\right.$$

and

(8) $$B_{n}^{\xi s}=\left\{\begin{array}{ccc} \sqrt{\frac{|\epsilon_{n}|-\mathrm{sign}\left(n\right)\Delta_{\xi s}}{2|\epsilon_{n}|}} & \;\mathrm{for}\; & n\neq 0\\ \frac{1+\xi }{2} & \;\mathrm{for}\; & n=0. \end{array}\right.$$

In turn, $\varphi_{n}\left(\cdot \right)$ is the Hermite function

(9) $$\varphi_{n}\left(y\right)=\frac{\omega^{1/4}}{\sqrt{2^{n}n!\sqrt{\pi }}}e^{-\omega y^{2}/2}H_{n}\left(\sqrt{\omega }y\right),$$

where $H_{n}\left(\cdot \right)$ is the Hermite polynomial of *n*-th order [27,28].

**Figure 2:**
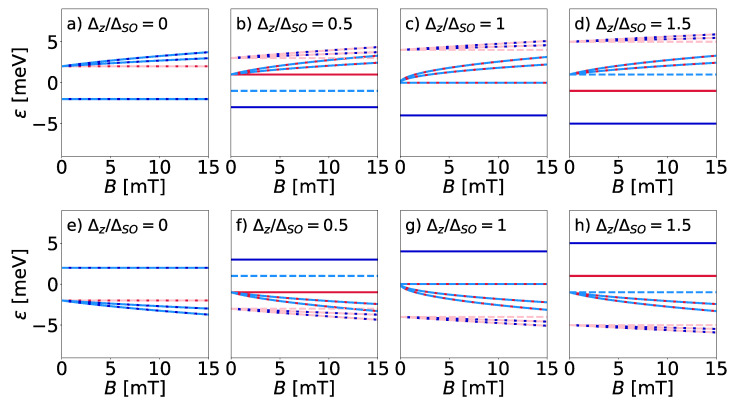
The dependence of the five lowest Landau levels n=0,±1,±2 on the magnetic field with different values of the applied electric field for the (**a**–**d**) electrons and (**e**–**h**) holes. The red and blue lines represent the spin-up and spin-down, respectively, while solid and dashed lines correspond to the K and $K^{\prime }$ valleys.

Figure 2 shows the five lowest Landau levels for analogous ratios to those presented before (cf. Figure 1) as a function of the magnetic field, *B*. Here, we distinguished the situation between electrons (Figure 2a–d) and holes (Figure 2e–h), which is important in further considerations of entropy. Thus, compared with the previous situation, the magnetic field causes the quantization of the energy bands into discrete Landau levels, labeled by the number *n*. The colors mark the spin-up (the red lines) and spin-down (the blue lines), while the line type denotes the K (the solid line) or the $K^{\prime }$ (the dashed lines) valleys. Figure 2a depicts a situation without an electric field, showing degenerated Landau levels for both valleys. The zeroth Landau levels remain constant, yet they do not lie at zero energy. The remaining levels scale with $\propto \sqrt{B}$. Such behavior remains in every plot, regardless of the value of the electric field, meaning a constant value for the n=0 and proportional to $\sqrt{B}$ for n≠0. In Figure 2b, as the electric field is non-zero, both the spin and valley degeneracy vanish for the zeroth level and partially disappear for n≠0. All the Landau levels shift up or down. The zeroth levels of spin-up electrons for the K valley and spin-down electrons for the $K^{\prime }$ valley are closer to each other, while the remaining two move away. These zeroth Landau levels have become degenerate, whereas the others continue to separate, as shown in Figure 2c. In Figure 2d, where the electric field is higher than the spin–orbit coupling, we can notice the band inversion between the zeroth Landau level of spin-up electrons for the K valley and spin-down electrons for the $K^{\prime }$ valley.

## 3. Phase-Space Approach

Let us note that the presented quantum-mechanical description of the silicon layer in the coordinate representation forces one to introduce a two-dimensional position operator $\hat{r}=\left(x,y\right)$ and a two-dimensional differential operator of momentum $\hat{p}=\left({\hat{p}}_{x},{\hat{p}}_{y}\right)$. These two operators form a pair of canonically conjugate variables, which can be further used to construct any Hermitian operators associated with some physical observables typical of the considered system. In turn, the phase-space formulation of the quantum theory based on Wigner’s approach utilizes the concept of the Weyl symbol of such operators. This quantity directly relates a Hermitian operator, $\hat{O}=O\left(\hat{r},\hat{p}\right)$, acting on the Hilbert space $L^{2}\left(R^{4}\right)$ with an ordinary function, $O_{W}\left(r,p\right)$, defined on the phase space, $R^{4}$, generated by the canonically conjugated variables (r,p). This mapping is realized by the inverse Weyl transform, which associates the matrix element of the considered Hermitian operator, $\hat{O}$, with the Weyl symbol of this one according to the formula

(10) $$O_{W}\left(r,p\right)=\int_{R^{2}}d^{2}R\;\langle r-\frac{1}{2}R|O\left(\hat{r},\hat{p}\right)|r+\frac{1}{2}R\rangle \exp\left(-\frac{i}{\hbar }p\cdot R\right),$$

where the Dirac notation is used. Applying this formula to the rescaled density operator, $\hat{\rho }=1/{\left(2\pi \hbar \right)}^{2}\left|\phi \rangle \langle \phi \right|$, for the pure states, |ϕ〉, allows one to introduce the Wigner distribution function (WDF) to describe these states within the phase-space approach as follows:

(11) $$\varrho \left(r,p\right)=\frac{1}{{\left(2\pi \hbar \right)}^{2}}\int_{R^{2}}d^{2}R\;\phi^{*}\left(r+\frac{1}{2}R\right)\phi \left(r-\frac{1}{2}R\right)\exp\left(-\frac{i}{\hbar }p\cdot R\right).$$

The WDF exhibits properties characteristic of the proper probability distribution function, with a notable exception: the WDF is negative in some regions of the phase space. Nonetheless, this property does not in any way disqualify the WDF as an effective calculating tool in practice. Let us note that the marginals of the WDF have the correct physical interpretation, i.e., they are well-defined probability densities in real and momentum spaces. The negativeness of the WDF is not also an obstacle to determining any expected values of dynamical variables because, for the Weyl symbols associated with them, these quantities are expressed by the phase-space integrals in the form

(12) $$\langle O\rangle =\int_{R^{4}}d^{2}rd^{2}p\;O_{W}\left(r,p\right)\varrho_{m}\left(r,p\right),$$

which gives correct values. Finally, the negativity of the WDF is often used as an indicator of the state quantumness [29]. In recent years, this last observation led to the discovery of an exact relationship between the nonclassical parameter and Wigner–Rényi one-half entropy [30]. It is essential to highlight that this result applies to the pure states [31]. One of the significant consequences resulting from this restriction is that the WDF can be regarded as the amplitude of the probability density in the phase space, and consequently, the square modulus of the WDF in the sense of the norm $L^{2}\left(R^{4}\right)$ gives the probability density in the phase space. According to this concept, the auxiliary function $\tilde{\varrho }\left(r,p\right)$ can be introduced:

(13) $$\tilde{\varrho }\left(r,p\right)=\frac{\varrho (r,p)}{{\left||\varrho \left(r,p\right)|\right|}_{L^{2}\left(R^{4}\right)}},$$

where ${\left||\varrho \left(\cdot ,\cdot \right)|\right|}_{L^{2}\left(R^{4}\right)}$ is the norm in $L^{2}\left(R^{4}\right)$. With it, we introduce the Wigner–Rényi entropy for the Rényi index α, as follows:

(14) $$S_{\alpha }=\frac{1}{1-\alpha }\ln\left[\int_{R^{4}}d^{2}rd^{2}p\;{\left(|\tilde{\varrho }{\left(r,p\right)|}^{2}\right)}^{\alpha }\right],$$

where 0<α<∞ and α≠1. The interesting property of the Wigner–Rényi entropy is its relation to Wigner–Rényi one-half entropy [30], Shannon entropy [18] and collision entropy [32] for the Rényi indices α=1/2,1,2, respectively.

## 4. Results

### 4.1. Wigner Distribution Function for Silicene in EM Field

According to the results presented in Section 2, the spin-dependent wave function of the *n*-th Landau states is given by Equation (6). It is important to note that this function has a two-component form. This observation is a direct reason for the extension of the previously presented definition of the WDF [cf. Equation (11)] to the matrix form. Given this, the matrix WDF for the two-component wave function $\chi^{T}\left(r\right)=\left[f\left(r\right),g\left(r\right)\right]$, where f(·),g(·) are square-integrable scalar functions and the symbol *T* denotes the transposition, can be written in the form

(15) $$\hat{\varrho }\left(r,p\right)=\frac{1}{{\left(2\pi \hbar \right)}^{2}}\int_{R^{2}}d^{2}R\;\chi^{†}\left(r+\frac{1}{2}R\right)\chi \left(r-\frac{1}{2}R\right)\exp\left(-\frac{i}{\hbar }p\cdot R\right)$$

or equivalently, we write down this expression in matrix form as follows:

(16) $$\hat{\varrho }\left(r,p\right)=\left[\begin{array}{cc} \varrho_{11}^{ff}\left(r,p\right) & \varrho_{12}^{fg}\left(r,p\right)\\ \varrho_{21}^{gf}\left(r,p\right) & \varrho_{22}^{gg}\left(r,p\right) \end{array}\right],$$

where the diagonal elements $\varrho_{ii}$, with i=1,2, of this matrix are WDFs of the pairs (f,f) and (g,g) in common sense and the off-diagonal elements $\varrho_{ij}$, with i≠j, are the cross-WDFs of the pairs (f,g) and (g,f). By substituting the two-component wave function $\psi_{n}\left(r\right)$, corresponding to the *n*-th Landau state given by Equation (6), into the above definition in (15), we obtain the matrix WDF $\hat{\varrho }\left(r,p\right)$ in the following form:

(17) $${\hat{\varrho }}_{n}\left(r,p\right)=\left[\begin{array}{cc} {\left(A_{n}^{\xi s}\right)}^{2}W_{n_{+},n_{+}}\left(y,p_{y}\right) & iA_{n}^{\xi s}B_{n}^{\xi s}W_{n_{-},n_{+}}\left(y,p_{y}\right)\\ -iA_{n}^{\xi s}B_{n}^{\xi s}W_{n_{+},n_{-}}\left(y,p_{y}\right) & {\left(B_{n}^{\xi s}\right)}^{2}W_{n_{-},n_{-}}\left(y,p_{y}\right) \end{array}\right]W_{{\tilde{p}}_{x}}\left(x,p_{x}\right).$$

Let us note that the elements of this matrix WDF have a product form, $W_{n_{\pm },n_{\pm }}\left(y,p_{y}\right)$$W_{{\tilde{p}}_{x}}\left(x,p_{x}\right)$, each of which depends solely on one set of the phase-space variables. The explicit form of these $W_{q}\left(\cdot ,\cdot \right)$-functions is as follows:

(18) $$W_{{\tilde{p}}_{x}}\left(x,p_{x}\right)=\delta \left(p_{x}-{\tilde{p}}_{x}\right),$$

and

(19) $$W_{n_{\pm },m_{\pm }}\left(y,p_{y}\right)=\frac{1}{\pi \hbar }\int_{R}dY\;e^{ip_{y}Y/\hbar }\varphi_{n_{\pm }}\left(y-Y/2\right)\varphi_{m_{\pm }}^{*}\left(y+Y/2\right),$$

where ${\tilde{p}}_{x}=\hbar {\tilde{k}}_{x}$ is a fixed value of the momentum along the *x*-axis and $n_{\pm }=\left|n\right|-\left(1\pm \xi \right)/2$ is the integer quantum number that suitably pertains to electrons or holes in one of the two inequivalent valleys K and $K^{\prime }$. The diagonal elements of the matrix WDF defined by Equation (17), namely, $\varrho_{n_{+},n_{+}}$ and $\varrho_{n_{-},n_{-}}$, correspond to the WDFs associated with the electrons of the energy $\epsilon_{n}$, which belong to the sublattices *A* and *B*, respectively. In turn, the off-diagonal elements of this matrix are given by the cross-WDFs, which are related to the quantum correlation phenomena and entanglement. The analysis of these terms and their consequences is beyond the scope of the present study. For further considerations, we reduced the matrix WDF given by Equation (17) to the scalar form by calculating its trace according to the formula [33,34,35]

(20) $$\varrho_{n_{\pm }}\left(r,p\right)=\mathrm{Tr}\left\{{\hat{\varrho }}_{n_{\pm }}\left(r,p\right)\right\},$$

where $\mathrm{Tr}\left\{\cdot \right\}$ denotes the trace over the sublattices. In consequence, we introduce the scalar WDF in the following form:

(21) $$\begin{array}{ccc} \varrho_{n_{\pm }}\left(r,p\right) & = & \frac{\delta (p_{x}-{\tilde{p}}_{x})}{\pi \hbar }e^{-\left(\frac{p_{y}^{2}}{m\hbar \omega }+\frac{m\omega y^{2}}{\hbar }\right)}\left[{\left(A_{n}^{\xi s}\right)}^{2}{\left(-1\right)}^{n_{+}}L_{n_{+}}\left(\frac{2p_{y}^{2}}{m\hbar \omega }+\frac{2m\omega y^{2}}{\hbar }\right)\right.\\ & + & \left.{\left(B_{n}^{\xi s}\right)}^{2}{\left(-1\right)}^{n_{-}}L_{n_{-}}\left(\frac{2p_{y}^{2}}{m\hbar \omega }+\frac{2m\omega y^{2}}{\hbar }\right)\right], \end{array}$$

where $L_{n}\left(\cdot \right)$ is the Laguerre polynomial of order *n* and the other symbols have the same meaning as before.

### 4.2. Combined Wigner–Rényi Entropy for Electrons and Holes

In the series of papers [36,37,38,39,40], the authors introduced various inspiring concepts of information-theoretic and statistical measures of characterizing quantum or topological phase transitions in two-dimensional systems, like silicene. Especially, one of the entropic measures considered by them is the relative Rényi entropy [41], which was used to define the topological quantum number in the following way [36]:

(22) $$C\left(\Delta_{z}\right)=\mathrm{sign}\left\{\frac{\partial R_{\alpha }^{n,s}\left(\Delta_{z}\right)}{\partial \Delta_{z}}\frac{\partial R_{\alpha }^{n,-s}\left(\Delta_{z}\right)}{\partial \Delta_{z}}\right\},$$

where $R_{\alpha }^{n,s}\left(\Delta_{z}\right)$ is a sum of the relative Rényi entropies for electrons and holes. The quantity $C(\Delta_{z})$ can be seen as the topological charge, which takes the values ±1, i.e., $C(\Delta_{z})=1$ for the bulk insulator and $C(\Delta_{z})=-1$ for the topological insulator. The relative Rényi entropies are defined in the following way [41]:

(23) $$R_{\alpha }^{n,s}=\frac{1}{\alpha -1}\;\ln\;\int_{R^{2}}d^{2}r\;{\left[\rho_{n}^{s}\left(r\right)\right]}^{\alpha }\;{\left[\rho_{0}^{s}\left(r\right)\right]}^{1-\alpha },$$

where $\rho_{n}^{s}\left(r\right)={\left|\psi_{n}^{s}\left(r\right)\right|}^{2}$ are the diagonal elements of the density matrix for the *n*-th state in position representation and $\rho_{0}\left(r\right)$ is the reference to the diagonal elements of the ground-state density matrix. Let us note that the relative Rényi entropies give a local comparison of the distribution functions in the real space, in this case the distance between position densities for the *n*-th and 0-th Landau states. On the other hand, this approach neglects the information contained in the off-diagonal elements of the density matrix, which are associated with correlations or coherence. In the present study, we propose calculating the topological quantum number with the aid of the auxiliary function, $\tilde{\varrho }\left(r,p\right)$, according to the procedure included at the end of Section 3. The crucial point of this approach is to replace the Rényi entropy in the formula given by Equation (22) by the Wigner–Rényi entropy expressed by Equation (14). In consequence, we can express the topological quantum number by the formula

(24) $$C\left(\Delta_{z}\right)=\mathrm{sign}\left\{\frac{\partial {\tilde{S}}_{\alpha }^{n,s}\left(\Delta_{z}\right)}{\partial \Delta_{z}}\frac{\partial {\tilde{S}}_{\alpha }^{n,-s}\left(\Delta_{z}\right)}{\partial \Delta_{z}}\right\},$$

where ${\tilde{S}}_{\alpha }^{n,\pm s}\left(\Delta_{z}\right)$ denotes the combined Wigner–Rényi entropy for electrons and holes for a given *n*-th Landau level. Its form is given by the sum of the Wigner–Rényi entropies for electrons and holes, respectively, as follows:

(25) $${\tilde{S}}_{\alpha }^{n,s}\left(\Delta_{z}\right)=S_{\alpha }^{n,s}\left(\Delta_{z}\right)+S_{\alpha }^{-n,s}\left(\Delta_{z}\right).$$

The introduced Wigner–Rényi entropy is sensitive to the position–momentum correlations, whereas the relative Rényi entropy is insensitive to them. In other words, the Wigner–Rényi entropy reveals additional information associated with the quantum state, namely, the position–momentum correlations that are completely invisible in the relative Rényi entropy defined based on the density in real space. This observation is essentially significant because the magnetic field introduces these types of correlations. Another advantage of the presented extension is the computational cost-effectiveness; namely, for a given wave function, the WDF is determined once, according to Equation (15), and then normalized in the sense of the norm in $L^{2}\left(R^{4}\right)$. The above transformations are carried out to construct the auxiliary function, whose form, according to the reinterpretation of the WDF, is used in the definition of the Wigner–Rényi entropy. Let us note that this treatment does not require introducing the Husimi function as a reference state into the calculation in order to ensure a well-defined entropy. It is also noteworthy to note that the Wigner–Rényi entropy for the Rényi index α=2 represents the collision entropy [31], which is related to the inverse participant ratio in the phase space [42]. This result seems especially important in the context of Refs. [37,38], where the authors characterize the topological–band insulator transitions in silicene using the inverse participation ratio. Consistent with the presented approach, this result can be obtained by the application of the Wigner–Rényi entropy for the Rényi index α=2 because this quantity is regarded as the logarithmic measure of the localization of the state in the phase space. According to this, we calculated the combined Wigner–Rényi entropy for the Rényi index α=2 to investigate the topological–band insulator transitions with the aid of this indicator. Finally, we also determine the Shannon entropy (α=1) and one-half entropy (α=0.5) according to the proposed approach.

**Figure 3:**
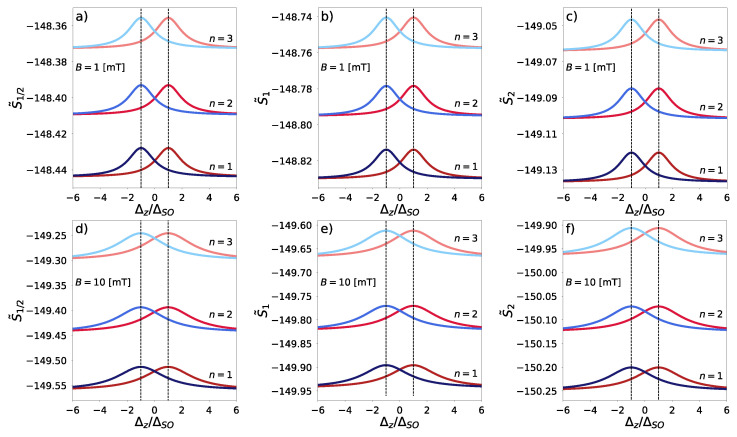
Combined Wigner–Rényi entropy for Landau states n∈{1,2,3}, and magnetic field, B=1 mT (**a**–**c**), and B=10 mT (**d**–**f**) for Rényi indices, α=1/2, (**a**,**d**) α=1, (**b**,**e**), α=2, (**c**,**f**). Red lines denote entropy for spin-up (s=1) state, while blue lines denote entropy for the spin-down (s=−1) state.

The results of such approach are demonstrated through the analysis of combined Wigner–Rényi entropy, as shown in Figure 3. The combined Wigner–Rényi entropy exhibits bell-shaped curves across Rényi indices α∈{0.5,1,2} and Landau levels n∈{1,2,3} for magnetic fields B=1 mT and B=10 mT. The maxima of these curves align with the critical point $\Delta_{z}/\Delta_{SO}=1$, where the system transitions from topological insulator to band insulator. Spin-up (red) and spin-down (blue) states display complementary antisymmetric profiles, providing the foundation for our topological quantum number definition. Higher magnetic fields result in more diffuse curves with increased broadening, similar to Gaussian distributions with larger standard deviations.

**Figure 4:**
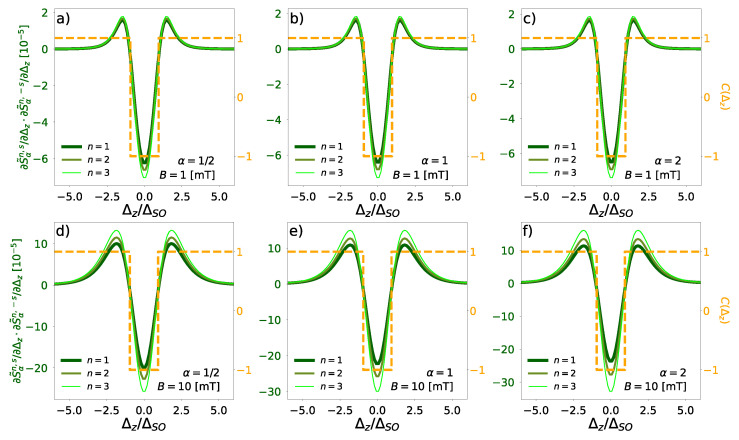
The green lines denote product of the derivatives of the combined Wigner–Rényi entropy for spin-up and spin-down states, for Landau states n∈{1,2,3}, and magnetic field B=1 mT (**a**–**c**) and B=10 mT (**d**–**f**) for Rényi indices, α=1/2, (**a**,**d**), α=1, (**b**,**e**), α=2 (**c**,**f**). Yellow line denotes sign of the product of these derivatives, which is topological quantum number based on combined Wigner–Rényi entropy.

The topological quantum number $C(\Delta_{z})$ derived from entropy derivatives is presented in Figure 4. Green curves show the product $\partial {\tilde{S}}_{\alpha }^{n,s}/\partial \Delta_{z}\cdot \partial {\tilde{S}}_{\alpha }^{n,-s}/\partial \Delta_{z}$, while yellow lines represent the sign of this product serving as our topological invariant. For all three Rényi indices, we are able to consistently identify the phase transition at $\Delta_{z}/\Delta_{SO}=1$, where $C(\Delta_{z})$ switches from −1 (topological insulator) to +1 (band insulator). For a higher magnetic field (B=10 mT), the derivatives become more separated for different Landau levels, with the Rényi entropy α=0.5 showing higher sensitivity to $\Delta_{z}/\Delta_{SO}$ variations. The Shannon (α=1) and collision (α=2) entropies exhibit progressively reduced sensitivity with higher amplitude across values. Nevertheless, all three calculated entropies yield the correct topological invariant under more intense magnetic fields.

## 5. Conclusions

We utilized the concept of the Wigner–Rényi entropy for the Rényi index α to investigate the influence of parallel magnetic and electric fields on topological phase transition in the silicene layer employing the topological quantum number. These results were obtained within the phase-space approach based on the Wigner distribution function, whose interpretation has been changed. In the presented approach, this function is treated as the wave function of a pure state in the phase-space representation. Hence, its square module represents the probability density in the phase space. This reinterpretation of the Wigner distribution function allows one to neglect the problem of its negativity. Simultaneously, it also allows one to define the concept of entropy based on this function. It is crucial to emphasize that the presented exact form of the Wigner distribution function has been derived from the two-component spin-dependent wave functions according to its standard definition. In the further part, we used the determined Wigner distribution function to calculate the Wigner–Rényi entropy for the Rényi indices α=1/2,1 and 2. We introduced the combined Wigner–Rényi entropies for these values of the Rényi indices. Finally, these quantities just served us in calculating the topological quantum number. The presented results demonstrate that the combined Wigner–Rényi entropy successfully identifies the topological phase transitions in silicene, with the characteristic bell-shaped curves exhibiting maxima at the critical point $\Delta_{z}/\Delta_{SO}=1$. All the three Rényi indices (α=0.5,1,2) correctly distinguish between topological insulator (C=−1) and band insulator (C=+1) states across different magnetic field strengths. The method is computationally efficient, requiring only the calculation of the Wigner distribution function. Such phase-space approach provides a robust tool for investigating topological quantum systems using information-theoretic measures in application to other two-dimensional gapped Dirac materials. The proposed entropic method of calculating the topological quantum number can be realized when applying the tomographical methods of reconstructing the Wigner distribution function [43,44,45,46,47]. We believe that this approach can be improved by deep machine learning, reducing the efforts needed to reconstruct the Wigner distribution function accurately.

## Data Availability

The original contributions presented in the study are included in the article, further inquiries can be directed to the corresponding author.

## Abbreviations

The following abbreviations are used in this manuscript:

WDF	Wigner distribution function
BI	Band insulator
TI	Topological insulator
VSPM	Valley–spin-polarized metal
TPT	Topological phase transition
